# Blockade of Exosome Release Sensitizes Breast Cancer to Doxorubicin via Inhibiting Angiogenesis

**DOI:** 10.1002/cam4.70785

**Published:** 2025-04-18

**Authors:** Jindi He, Fengyi He, Qinlian Yang, Qiuyun Li

**Affiliations:** ^1^ Department of Breast Surgery Guangxi Medical University Cancer Hospital Nanning Guangxi China

**Keywords:** Adriamycin, angiogenesis, breast cancer, exosome, GW4869

## Abstract

**Background:**

Chemotherapy combined with angiogenesis inhibition holds great promise in improving the therapeutic efficacy in cancer treatment. The aim of this study was to explore the effect of exosome blockade on tumor angiogenesis and chemotherapy efficacy.

**Methods:**

Exosomes were extracted by ultracentrifugation, and the effect of exosomes on angiogenesis was evaluated by 4T1 mouse breast cancer cell line and the syngeneic mouse tumor model and immunofluorescence. The endocytosis of exosomes from vascular endothelial cells was evaluated in vitro by co‐culture and immunofluorescence assays. Tube formation and CCK‐8 assays were used to evaluate the effect of exosomes on angiogenesis in vitro. The effect of exosome blockade on the efficacy of doxorubicin was evaluated by 4T1 mouse breast cancer model, cancer cell‐derived exosomes (Exo^4T1^), GW4869 and doxorubicin in vivo.

**Results:**

Exo^4T1^ can be efficiently endocytosed by vascular endothelial cells both in vitro and in vivo. Within the recipient endothelial cells, Exo^4T1^ elicited angiogenesis at least partially via promoting cell proliferation, as the exosomes were carrying cargos with pro‐proliferation capacity. Blockade of exosome release through GW4869 significantly inhibited angiogenesis, increased the concentration of doxorubicin within the tumor, and sensitized the tumor to doxorubicin in the murine 4T1 syngeneic model, whereas the therapeutic effects were abrogated when Exo^4T1^ was additionally treated. Moreover, we found there was no synergy between GW4869 and pazopanib (PP, a traditional angiogenesis inhibitor).

**Conclusions:**

Together, we here revealed that cancer‐derived exosomes promote angiogenesis during cancer progression and GW4869 treatment would sensitize the cancer cells to doxorubicin at least partially via inhibiting angiogenesis.

## Graphical abstract

We here revealed that cancer‐derived exosomes promote angiogenesis during cancer progression, and GW4869 treatment would sensitize the cancer cell to doxorubicin mainly by inhibiting angiogenesis.

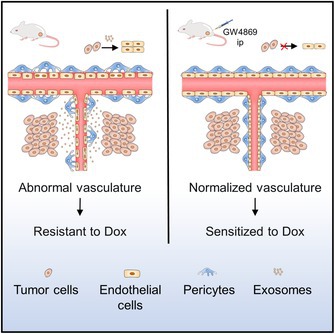

## 1 Introduction

Breast cancer stands as one of the most prevalent cancers globally. According to the latest available data from 2020, there were approximately 2.26 million new cases reported worldwide [1]. With the continuous development of surgical therapy, chemotherapy, radiotherapy, endocrine therapy, and targeted therapy have significantly enhanced survival rates. Nevertheless, challenges persist, particularly in combating chemotherapy resistance and managing side effects, especially in triple‐negative breast cancer (TNBC), which comprises 15%–20% of all cases [2].

Angiogenesis is an important feature of breast cancer [3], and anti‐angiogenesis holds great promise in tumor therapy [4, 5, 6]. Anti‐angiogenesis drugs inhibit the generation of tumor blood vessels, block the blood flow and nutrient supply of tumor tissues, and tumor cells are “starved to death.” In addition, antiangiogenic drugs restore normal tumor blood vessels, improve the sensitivity of tumor tissue to chemotherapy and reduce the metastasis of tumor cells [7]. Chemotherapy combined with anti‐angiogenesis therapy has been demonstrated to effectively inhibit tumor progression and metastasis [8, 9, 10]. And elucidating the specific mechanisms of angiogenesis in tumor tissues is beneficial to develop new strategies for anti‐angiogenesis therapy.

Exosomes are extracellular vesicles characterized by a diameter ranging from 40 to 160 nm, which can deliver proteins, lipids, mRNA, miRNA, lncRNA, and other nucleic acids to cells near or far away [11, 12, 13, 14, 15, 16]. Exosomes from tumor cells are crucial for communicating between cells and boosting tumor growth [17, 18, 19, 20, 21].

In this study, we confirmed that tumor cell‐derived exosomes promote tumor progression by promoting angiogenesis, whereas the exosome inhibitor GW4869 (GW) effectively improves the therapeutic effects of doxorubicin (Dox). The combination therapy of Dox and GW significantly inhibits the progression of tumors and is expected to serve as a promising combination therapy to improve therapeutic efficacy.

## 2 Material and Methods

### 2.1 Experimental Model and Subject Details

#### 2.1.1 Mice

The animal experiments conducted in this study received approval from the Institutional Animal Experiment Administration Committee of the Fourth Military Medical University. Female BALB/c mice at age 5–6 weeks were obtained from the Lab Animal Center of the Fourth Military Medical University. Approximately 5 × 10^5^ 4T1 cells were injected into the right fourth mammary fat pad of the BALB/c mice. Tumor volumes were measured and calculated according to the formula volume = length × width^2^ × 1/2. When the tumor volume was palpable, the mice in the group that combined GW or PP were pretreated with GW4869 (GW, 2.5 mg/kg, Sigma, D1692) through intraperitoneal injection three times a week, or pazopanib (PP, 30 mg/kg, Beyotime, SC0218) through intragastric administration every day for a week. Then continue the treatment as groups of PBS, Dox, Dox + DMSO, Dox + GW, Dox + GW + Exo^HC11^, Dox + GW + Exo^4T1^, Dox + PP, or Dox + GW + PP for another week. Doxorubicin (Dox, 8 mg/kg, Abmole, M1969) and exosomes (200 μg/mouse) were administered through intraperitoneal injection. After 2 weeks of treatment, mice were euthanized through an overdose of Sodium pentobarbital (150 mg/kg, intraperitoneal injection). Tumors and other tissues of interest were harvested for the next study. To determine the survival time after different treatments, animals were allowed to live until natural death.

#### 2.1.2 Cell Culture

Human Umbilical Vein Endothelial Cells (HUVECs) were purchased from iCell (iCell‐h110), mouse mammary epithelium HC11 was purchased from Procell (HC11), and breast cancer cell line 4T1 was purchased from FuHeng BioLogy (FH1114). HUVECs were routinely cultured in DMEM (GIBCO, C11995500BT) supplemented with 1% penicillin/streptomycin (Solarbio, 31800) and 10% fetal bovine serum (FBS, ExCell Bio, FND500), and HC11 and 4T1 cells were cultured in RPMI‐1640 (GIBCO, C11330500BT) supplemented with 1% penicillin/streptomycin and 10% FBS. All cells were cultured in an incubator at 37°C and in an atmosphere humidified with 5% CO_2_.

### 2.2 Method Details

#### 2.2.1 Exosome Isolation and Characterization

Exosomes were isolated from the conditioned medium of cells or tumor tissues by ultracentrifugation. Briefly, tumor tissues resected were minced into small pieces of 1 × 1 × 1 mm^3^, serum‐free medium was used to culture for 48 h [22], and then the supernatant was collected to isolate exosomes through ultracentrifugation. When cell fusion reached 80%, a serum‐free medium was used for continued culture; after 48 h, the conditioned medium was collected and centrifuged at 3000 *g* for 15 min and then 10,000 *g* for 30 min at 4°C to remove cells and cell debris, respectively. The supernatant was then gone through ultracentrifugation at 100,000 *g* for 70 min, and pellets were resuspended into one to ultracentrifuged again at 4°C, and the final purified exosomes were dissolved in Phosphate Buffered Saline (PBS) and stored at −80°C for use. The isolated exosomes were sent to ZetaView (Particle Metrix, USA) to measure the particle size distribution using nanoparticle tracking analysis (NTA). Transmission electron microscopy (TEM) was used to characterize the morphology and size of the isolated exosomes.

#### 2.2.2 Exosome Fluorescent Labeling and Tracking In Vivo/In Vitro

Purified exosomes were labeled using the DiI red fluorescent probe (Beyotime, C1036) or DiR (Invitrogen, D12731) according to the manufacturer's protocol. Briefly, a certain amount of exosomes was resuspended in PBS and mixed with DiI/DiR in a volume ratio of 500:1, incubated at 37°C for 5 min, and then at 4°C for 15 min. The labeled exosomes were then washed twice with PBS and co‐cultured with HUVECs (80 μg exosomes) or injected into tumor‐bearing BALB/c mice through the tail vein (200 μg exosomes/mouse). After 3 h of co‐culturing with HUVECs, cells were fixed with 4% paraformaldehyde for 15 min, and nuclei were stained by Hoechst 33258 (Beyotime, C1018) at room temperature for 5 min in the dark. Tumor and other tissues of interest were harvested 12 h after intravenous injection, and every tissue was cut into sections of 8 μm thickness with a cryostat (Leica, Germany), and the slices were fixed with 4% paraformaldehyde for 15 min, then nuclei were stained by Hoechst 33258. All pictures were taken with a confocal microscope (Nikon, Japan).

#### 2.2.3 Western Blot

The total protein of exosomes was extracted by RIPA Lysis Buffer (Beyotime, P0013B) added with 1% PMSF (Beyotime, ST506) as per the manufacturer's instruction. The protein concentration was determined utilizing the BCA Protein Assay Kit (Beyotime, P0012S). Equal amounts of protein underwent sodium dodecyl sulfate‐polyacrylamide gel electrophoresis (SDS‐PAGE) and subsequent transfer onto a Nitrocellulose (NC) membrane (biosharp, 66485). After blocking with 5% non‐fat milk for 1 h at room temperature, the membrane was incubated overnight at 4°C with the primary antibodies of GM130 (1:5000, Proteintech, 66662‐1‐Ig), CD81 (1:2000, Proteintech, 27855‐1‐AP), and TSG101 (1:5000, Proteintech, 67381‐1‐Ig). Following three washes in TBST, the membrane was then incubated with HRP‐conjugated secondary antibodies (Proteintech) for 1 h at room temperature. Chemiluminescent signals were captured using an imaging system (GE Healthcare, Chalfont St. Giles, UK) within a dark environment.

#### 2.2.4 Immunofluorescence

Tumor and other tissues of interest were harvested; every tissue was cut into sections of 8 μm thickness with a cryostat and was fixed with 4% paraformaldehyde for 15 min, permeabilized with 0.1% Triton X‐100 for 20 min, and after incubation with 5% BSA for 1 h at room temperature, the sections were incubated with the primary antibodies of anti‐CD31 (1:800, CST, 3528S) or anti‐Ki67 (1:500, Servicebio, GB111141) overnight at 4°C in a wet and dark box. After washing three times with PBS, the samples were incubated with the corresponding secondary antibody (Proteintech) at room temperature for 1 h in the dark, and nuclei were detected by treatment with Hoechst 33258. Pictures were taken with a confocal microscope.

#### 2.2.5 CCK‐8 Assay

Cell proliferation was assessed using the Cell Counting kit‐8 (CCK‐8, Servicebio, G4103). HUVECs were seeded in 96‐well plates at a density of 2000 cells in 100 μL of medium per well 24 h prior to exosome (40 μg or 80 μg) treatment. HUVECs were co‐cultured with different exosomes as indicated. At designated time points, CCK‐8 solution was added and incubated at 37°C in the dark for 1.5 h. Absorbance at 450 nm was determined utilizing a microplate reader (BioTek, USA).

#### 2.2.6 Tube Formation Assay

HUVECs (3 × 10^4^ cells) treated with PBS, 40 μg Exo^HC11^, 80 μg Exo^HC11^, 40 μg Exo^4T1^, and 80 μg Exo^4T1^ were seeded on 96 well plates, which had previously been coated with Matrigel (corning, 354248) After 4–6 h of incubation, tube‐like structures were observed under a microscope, and pictures were collected. Tube formation was quantified by determining the number of branching points using ImageJ.

#### 2.2.7 TUNEL Staining

TUNEL staining was conducted utilizing the One Step TUNEL Apoptosis Assay Kit (Beyotime, C1086) following the manufacturer's guidelines to detect tumor cell apoptosis.

#### 2.2.8 Measurement of Serological Parameters

Serological parameters creatine kinase‐MB (CK‐MB), blood urea nitrogen (BUN), alanine transaminase (ALT), and aspartate transaminase (AST) were measured using an automatic chemistry analyzer (Beckman Coulter, USA) according to the manufacturer's instructions.

#### 2.2.9 Quantification and Statistical Analysis

Data were expressed as mean ± S.D. Statistical evaluation of different groups was analyzed by one‐way analysis of variance (ANOVA); two‐way ANOVA was used to calculate tumor volume progression over time and the log‐rank test for survival curves. All statistical calculations were performed using GraphPad Prism 8.0.

## 3 Results

### 3.1 Tumor Cell‐Derived Exosome Biogenesis Increases During Tumor Angiogenesis

**FIGURE 1:**
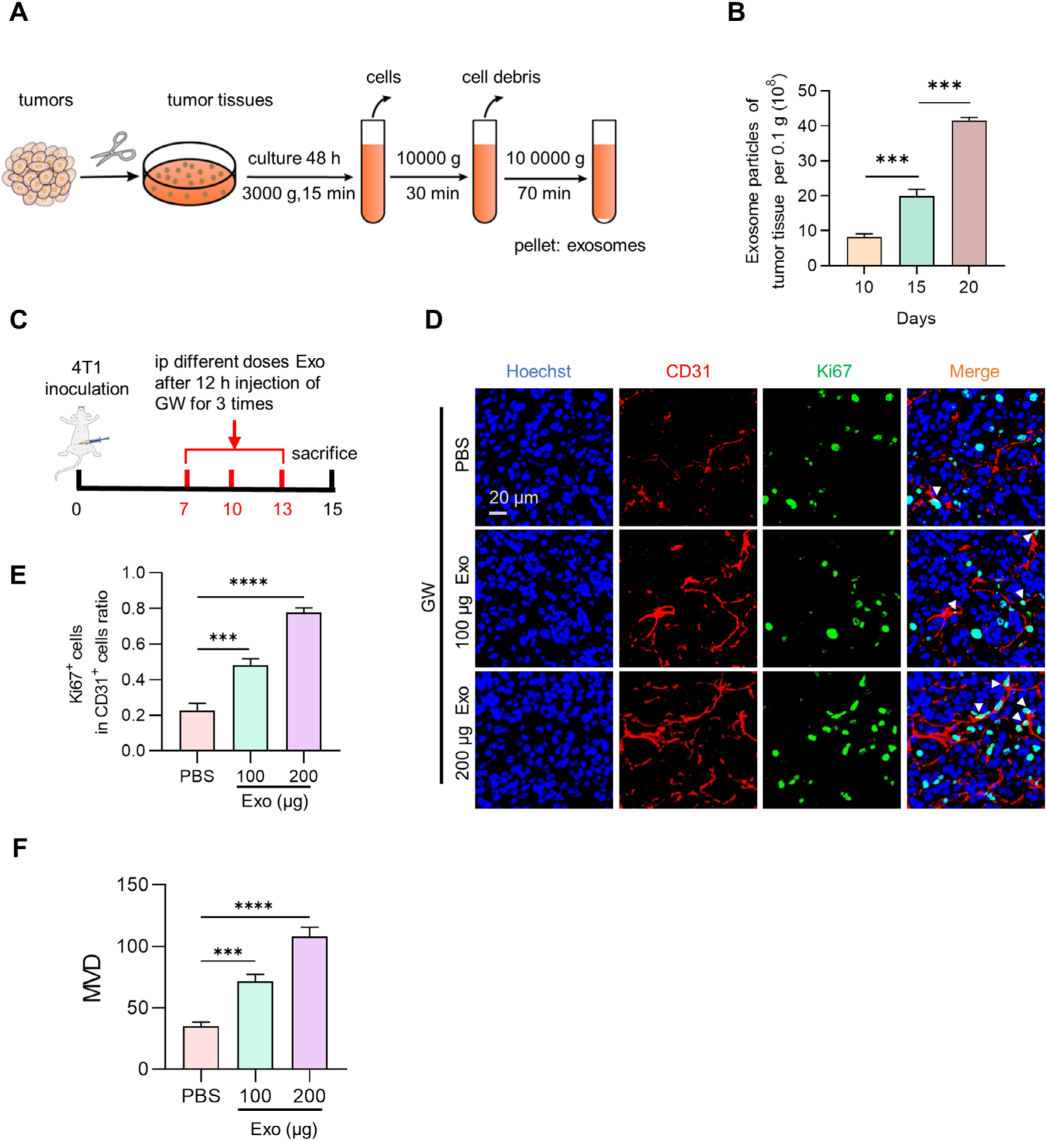
Tumor cell‐derived exosome biogenesis increases during tumor angiogenesis. (A) Isolation process diagram of exosomes from tumor tissue. Tumor tissues were minced and cultured with a serum‐free medium for 48 h, and then exosomes were purified as indicated. (B) Exosomes production per 0.1 g tumor tissue on day 10, 15, and 20 after 4T1 tumor cells inoculation. *n* = 3. (C) Schematic diagram of treatment way of (D). (D) Representative immunofluorescence images showing the co‐localization of CD31 (red) and Ki67 (green) in different exosome treatment groups. (E) Statistical analysis of (D), *n* = 3. (F) Statistical analysis of microvessel density (MVD) (number/visual field), *n* = 3. ***p < 0.001, ****p < 0.0001 by one way ANOVA.

Tumor cell‐derived exosomes, which possess nanoscale dimensions, good biocompatibility, and great contents, play a crucial role in facilitating communication among cells within the tumor microenvironment. Tumor‐bearing female BALB/c mice were sacrificed on days 10, 15, and 20, respectively after inoculation with 4T1 cells, and tumors in the different periods were resected to extract exosomes by ultracentrifugation (Figure 1A). With tumor progression, the production of tumor exosomes increased (Figure 1B). To investigate the effects of tumor exosomes on tumor angiogenesis, tumor‐bearing female BALB/c mice were first treated with GW4869 to exclude the influence of endogenous exosomes, followed by the injection of different doses of exosomes from the mouse breast cancer cell line 4T1 (Exo^4T1^). As expected, in the group treated with tumor cell‐derived exosomes, there were more CD31^+^ cells (Figure 1C–F), suggesting that tumor cell‐derived exosomes might promote tumor progression through angiogenesis.

### 3.2 Endocytosis of Tumor Cell‐Derived Exosomes by Vascular Endothelial Cells In Vitro and In Vivo

**FIGURE 2:**
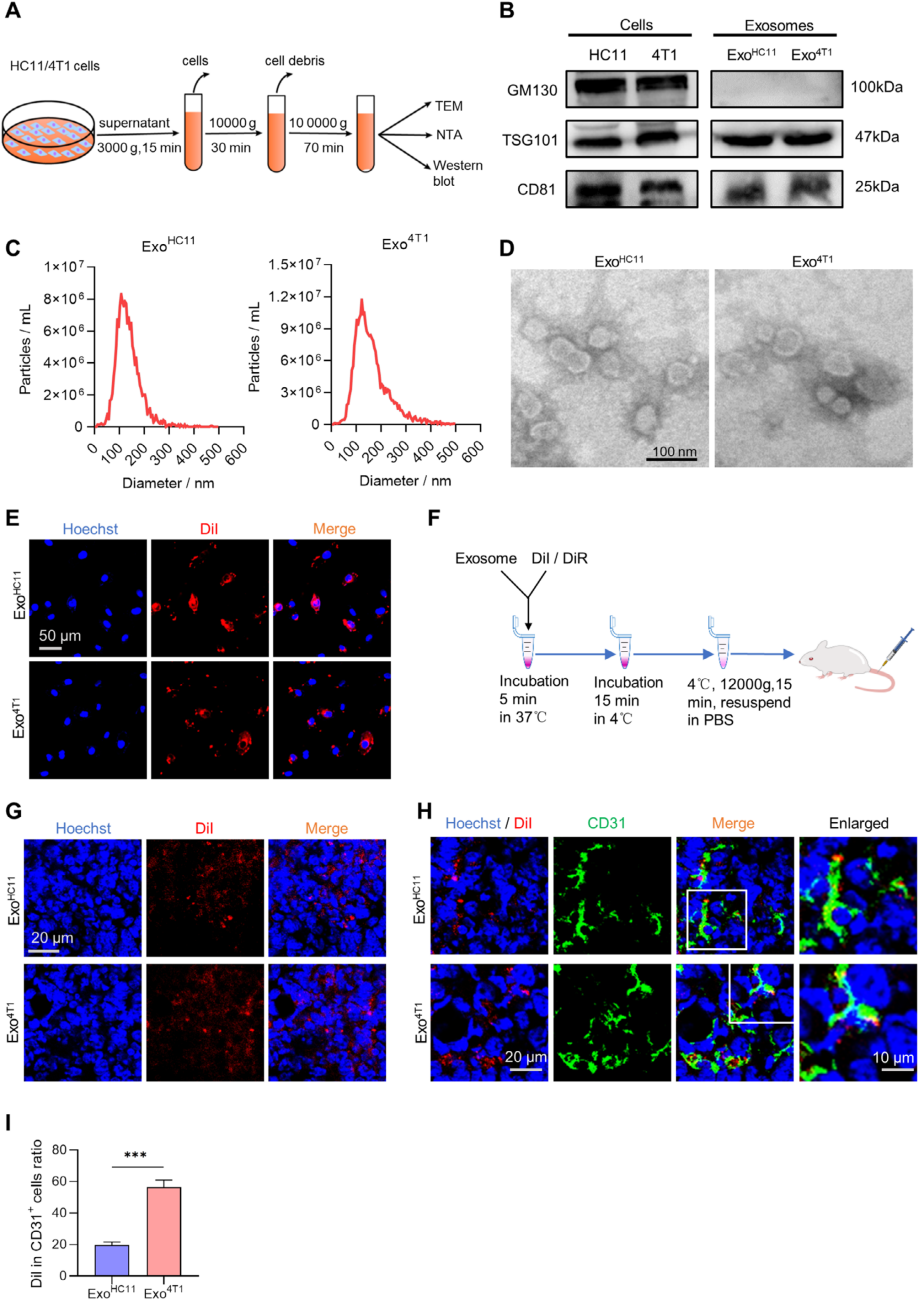
Endocytosis of tumor cell‐derived exosomes by vascular endothelial cells in vitro and in vivo. (A) Schematic diagram of exosome isolation and identification. (B) Western blot analysis of the expression of TSG101, CD81, and GM130 in cell lysates and exosomes. (C) Nanoparticle tracking analysis of collected exosomes. (D) Transmission electron microscopy (TEM) analysis of collected exosomes. (E) Representative microscopy images of the uptake of DiI labeled exosomes by HUVECs. Three independent experiments were performed and representative images are shown. (F) Diagram of exosome staining by DiI/DiR. (G) Representative microscopy images of the distribution of DiI labeled exosomes in the tumor. (H) Representative immunofluorescence images of DiI labeled exosomes (red) uptake by vascular endothelial cells (green) in vivo. *n* = 3. (I) Statistical analysis of (H). *** *p* < 0.001 by Student's t test.

We thus explored whether tumor‐derived exosomes can be endocytosed by endothelial cells. Exosomes from mouse breast cancer cell 4T1 (Exo^4T1^) were thus collected, and exosomes from normal mouse mammary epithelial cells HC11 (Exo^HC11^) served as the control (Figure 2A). Purified exosomes underwent characterization through Western blot analysis, Nanoparticle Tracking Analysis (NTA), and transmission electron microscopy (TEM). Western blot analysis confirmed the presence of exosome markers TSG101 and CD81, with the absence of GM130 (a marker for the cis‐Golgi compartment) in the isolated exosomes (Figure 2B). NTA and TEM analysis confirmed the size (40–160 nm diameter) and saucer‐like bilayer structure morphology of exosomes (Figure 2C,D).

HUVECs were then incubated with DiI‐labeled Exo^4T1^ or Exo^HC11^ for 3 h, and robust red fluorescence was found around nuclei, indicating efficient uptake of exosomes by HUVECs (Figure 2E). To explore the distribution of tumor cell‐derived exosomes in vivo (Figure 2F), ex vivo imaging analysis and confocal laser scanning microscope analysis were performed. Exo^4T1^ and Exo^HC11^ were found enriched in the tumors (Figure 2G). Notably, exosomes were also enriched in the liver, spleen, and lung (Figure S1A,B), and the distribution of Exo^4T1^ and Exo^HC11^ had no significant difference in vivo (Figure S1C). To further determine whether tumor cell‐derived exosomes could be endocytosed by the endothelial cells, co‐localization of endothelial cells and DiI‐labeled exosomes was examined. The results showed that tumor cell‐derived exosomes could be efficiently taken up by endothelial cells (Figure 2H,I).

### 3.3 Tumor Cell‐Derived Exosomes Promote Endothelial Cell Proliferation

**FIGURE 3:**
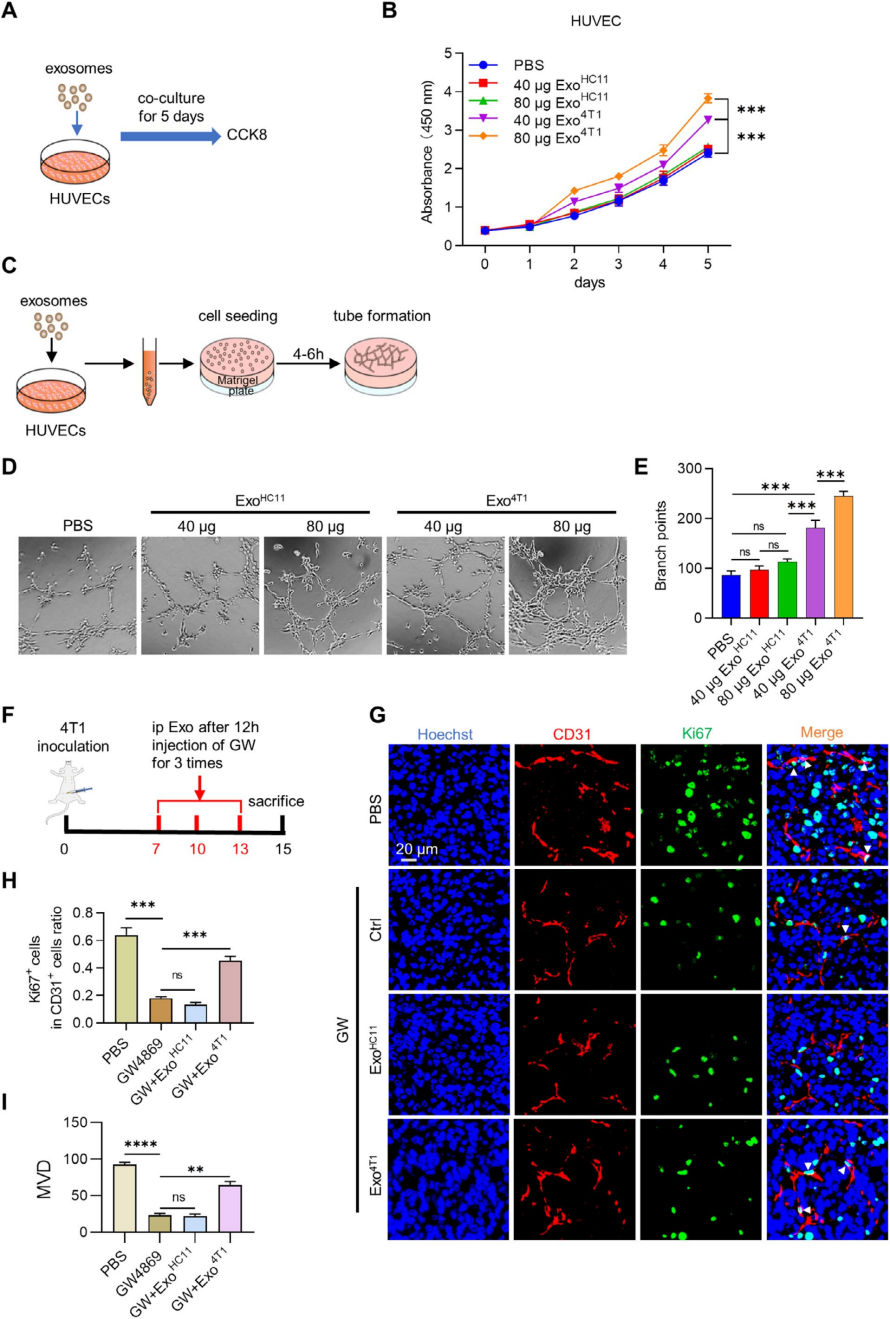
Tumor cell‐derived exosomes promote endothelial cell proliferation. (A) Schematic diagram of the co‐culture of exosomes and HUVECs. (B) The relative proliferation rate of HUVECs after treatment as indicated was analyzed by CCK8 assay. (C) Schematic diagram of the tube formation assay. (D) Tube formation was performed to detect the angiogenesis of HUVECs as indicated in vitro. (E) Quantitative analysis of tube formation. (F) Schematic diagram of treatment way of (G). (G) Representative images of immunostaining of Ki67 (green) and CD31 (red) in tumors after indicated treatments. *n* = 3. (H, I) Quantitative analysis of (G), three independent experiments were performed and data was shown as mean ± SD. **p < 0.01, ***p < 0.001, ****p < 0.0001 by one way ANOVA.

As tumor‐derived exosomes are pro‐proliferating, we next explored the effects of Exo^4T1^ on endothelial cell proliferation. HUVECs were thus treated with exosomes, and the proliferation of HUVECs was detected every day by using CCK8 (Figure 3A). Compared with the Exo^HC11^, Exo^4T1^ promoted the proliferation of HUVECs in a dose‐dependent manner (Figure 3B). Tube formation assay further confirmed that Exo^4T1^ significantly promoted angiogenesis (Figure 3C–E). To further confirm the effects in vivo, GW4869 was intraperitoneally injected into the tumor‐bearing female BALB/c mice 12 h to block the secretion of endogenous exosomes, followed by exosomes injection (200 μg/mouse) (Figure 3F). As expected, tumor angiogenesis was significantly decreased when tumor cell‐derived exosomes were blocked by GW4869, whereas additional treatment with tumor cell‐derived exosomes restored the angiogenesis (Figure 3G–I), suggesting that exosomes derived from tumor cells may enhance angiogenesis.

### 3.4 Tumor‐Cell‐Derived Exosomes Reduce the Therapeutic Efficacy of Doxorubicin

**FIGURE 4:**
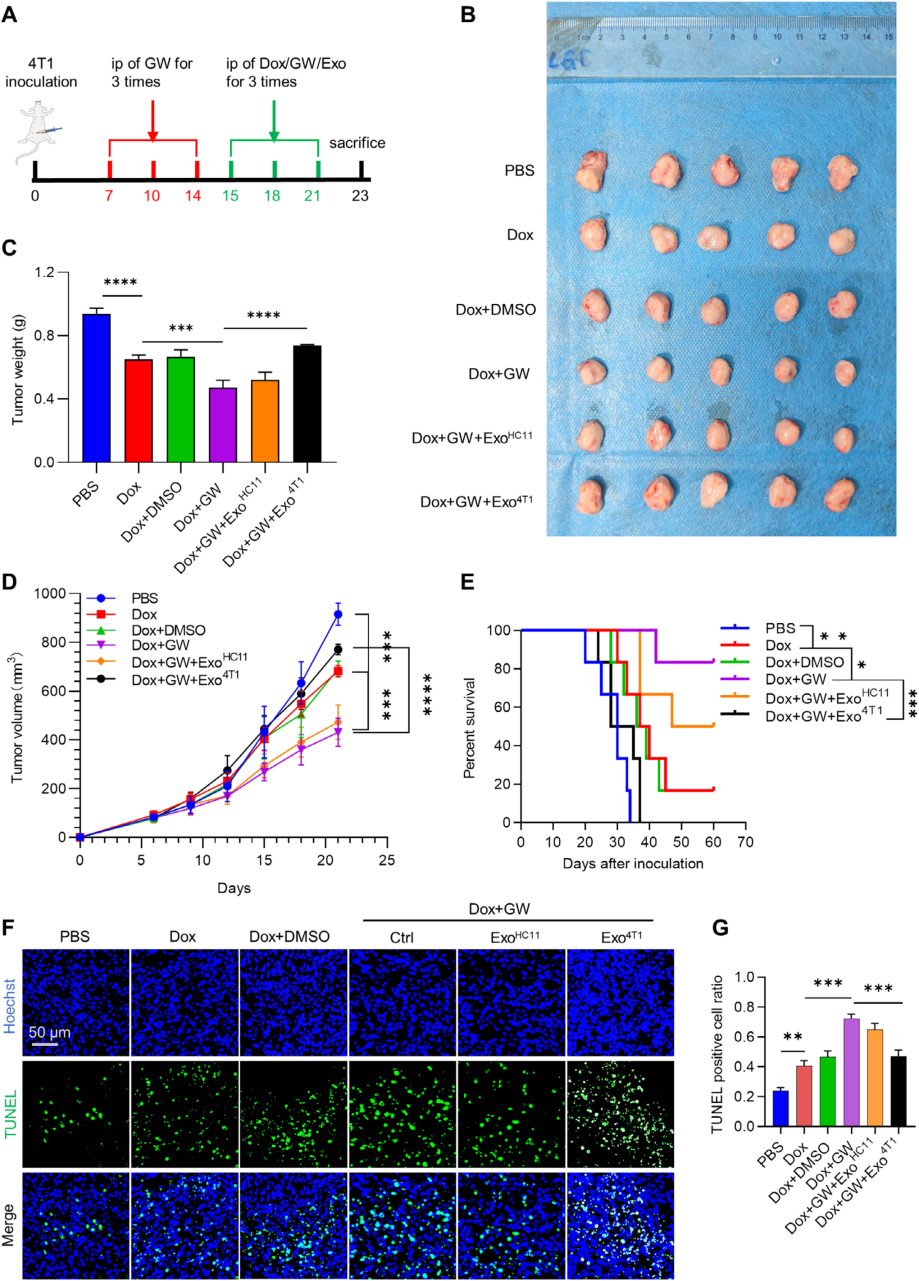
Tumor‐cell derived exosomes reduce the therapeutic efficacy of doxorubicin. (A) Schematic diagram of treatment schedule. (B) Image of excised tumors at the conclusion of the experiment. (C) Tumor weight at the end of the experiment. ***p < 0.001 by one way ANOVA. (D) Tumor volume progression as a function of time. Tumor size was monitored every 2 days. Data was expressed as mean ± SD; *n* = 5. ***p < 0.001 by two way ANOVA. (E) Survival curves of tumor‐bearing mice treated with PBS, Dox, Dox + DMSO, Dox + GW, Dox + GW + Exo^HC11^, and Dox + GW + Exo^4T1^ respectively. *n* = 5. (F) Representative TUNEL staining images of tumors from mice with indicated treatments. *n* = 5. (G) Quantitative analysis of (F). *p < 0.05, **p < 0.01, ***p < 0.001, ****p < 0.0001 by one way ANOVA.

Angiogenesis is closely related to drug delivery and chemotherapy resistance. We next explored the effects of tumor cell‐derived exosomes on breast cancer chemotherapy efficacy in a 4T1 breast cancer syngeneic mouse model (Figure 4A). Dox treatment alone significantly inhibited tumor growth, and the additional combination of GW4869 further suppressed tumor growth. The facilitated effect of GW4869 was abrogated by the injection of exogenous tumor cell‐derived exosomes (Figure 4B–D). In accordance with tumor suppression, the combination of Dox and GW4869 had the longest overall survival (Figure 4E). Mechanistically, the combination of GW4869 sensitized tumor cells to the apoptosis efficiency of Dox, whereas Exo^4T1^ treatment abrogated this effect (Figure 4F,G).

### 3.5 GW4869 Enhances Chemotherapy Efficacy by Increasing Doxorubicin Concentrations

**FIGURE 5:**
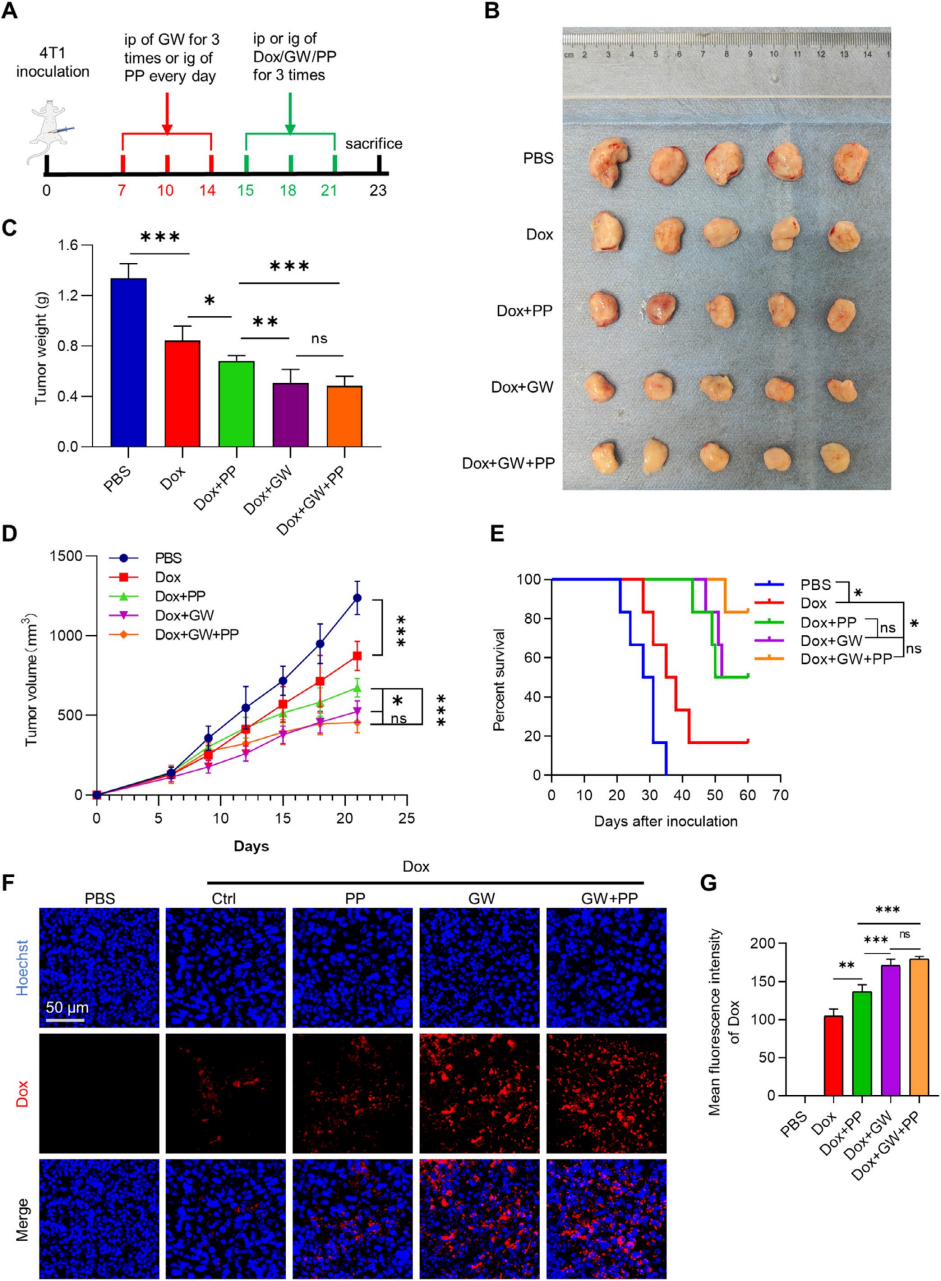
GW4869 enhances chemotherapy efficacy by increasing doxorubicin concentrations. (A) Schematic diagram of treatment schedule. (B) Image of excised tumors at the conclusion of the experiment. (C) Tumor weight at the conclusion of the experiment. (D) Tumor volume progression as a function of time. Tumor size was monitored every 2 days. Data were expressed as mean ± SD; *n* = 5. ***p < 0.001 by two way ANOVA. (E) Survival curves of tumor‐bearing mice treated with PBS, Dox, Dox + GW, Dox + PP, and Dox + GW + PP respectively, *n* = 5. (F) Representative confocal images of Dox distribution in tumors with indicated treatment. (G) Quantitative analysis of (F). *p < 0.05, **p < 0.01, ***p < 0.001 by one way ANOVA.

Past studies have shown that GW4869 inhibits tumor cell proliferation, migration, and invasion; EMT; and chemotherapy drug resistance in vitro [23, 24]. To find out whether GW4869 suppresses tumor growth by inhibiting angiogenesis directly, 4T1 breast cancer syngeneic mice were randomly divided into five groups: PBS, Dox, Dox + GW, Dox + PP, and Dox + GW + PP groups. PP, pazopanib, is an antiangiogenic agent by inhibiting VEGFR. As expected, both Dox + GW and Dox + PP treatment inhibited tumor growth and prolonged the survival time significantly, whereas Dox + GW had a more significant tumor suppression effect than Dox + PP. Notably, GW4869 and PP have no synergistic effects (Figure 5A–E). Consistent with the observed therapeutic effects, additional treatment of GW4869 or PP significantly increased the drug delivery of Dox into tumor tissues, whereas no synergistic effects of GW4869 and PP were observed (Figure 5F,G). Moreover, the addition of GW did not significantly increase the toxicity in the heart, liver, and kidney in mice (Figure S2A–D).

## 4 Discussion

**FIGURE 6:**
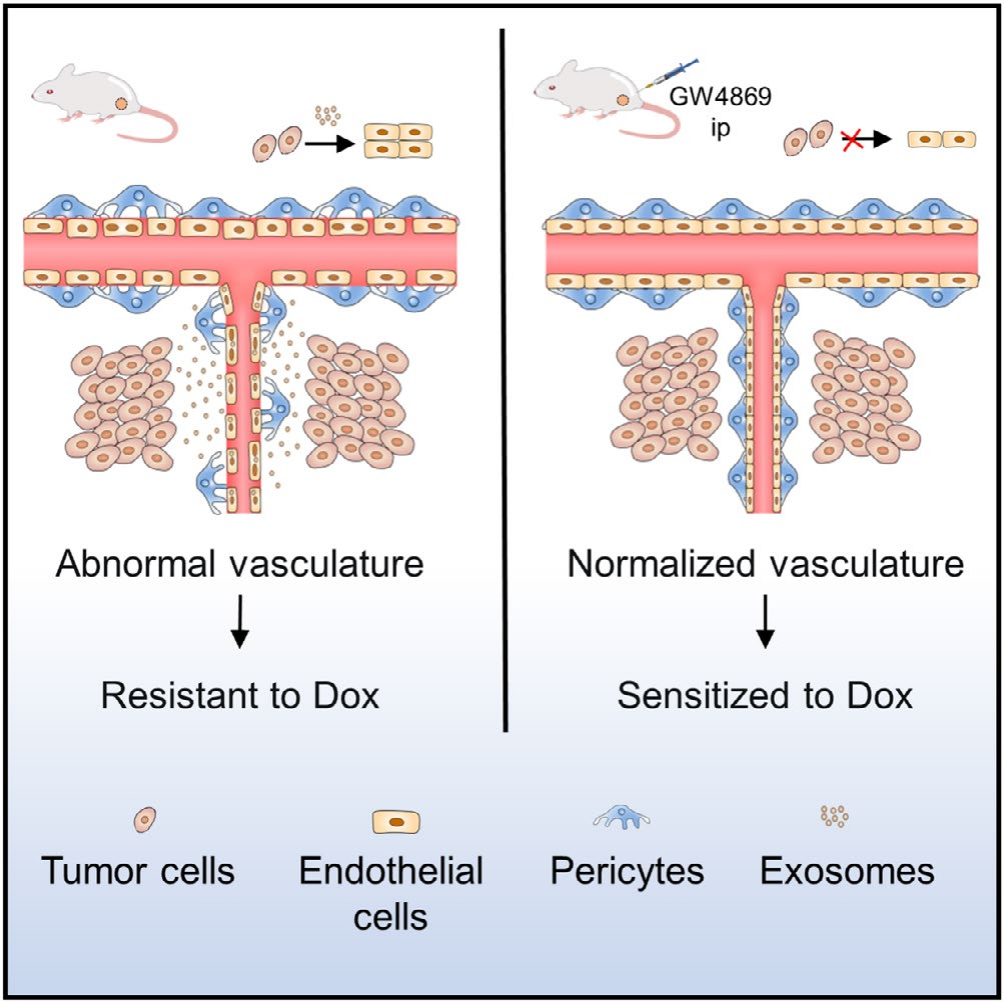
Schematic representation of the study findings. Tumor cell‐derived exosomes contribute to tumor progression by promoting angiogenesis, and blocking exosome secretion improves the chemotherapy effects in breast cancer model.

Angiogenesis is pivotal in the progression of tumors and is thought to be driven mainly by hypoxia and VEGF [3, 25, 26]. In this study, we demonstrated that tumor cell‐derived exosomes contribute to tumor progression by promoting angiogenesis, and blocking exosome secretion improves the chemotherapy effects in a breast cancer model (Figure 6).

Doxorubicin, an anthracycline drug that kills tumor cells by destroying their DNA structure, is commonly used in breast cancer chemotherapy [27]. Combination therapy is commonly used in clinical treatment, especially in the systemic treatment of chemotherapy [28, 29, 30, 31]. Given the intricacies of tumors, it is imperative to underscore the significance of employing combination therapy involving antitumor drugs with distinct mechanisms and targets. Effective combination therapy not only improves efficacy and reduces adverse reactions but also brings more treatment options for patients. Due to the abnormal angiogenesis of tumor tissues, a large number of vessels with abnormal function seriously affect the penetration of antitumor drugs into tumor tissues [32]. Combination therapy regimens of different anti‐angiogenesis drugs and various antitumor drugs have been continuously explored, and some of them have been used clinically. Current anti‐angiogenesis therapeutic strategies mainly focus on blocking VEGF/VEGFR and the upstream or downstream molecules [33, 34, 35]. Therapeutic drugs are mainly divided into two categories: large molecular monoclonal antibodies with a single target, such as bevacizumab and ramucirumab, and small molecular inhibitors with multiple targets, such as sorafenib, apatinib, and pazopanib [36]. We here demonstrated that tumor cell‐derived exosomes promote tumor angiogenesis at least partially via cell proliferation promotion, and exosome inhibitor GW4869 inhibits angiogenesis. Moreover, combined Dox and GW therapy significantly improved the therapeutic efficacy, suggesting that Dox + GW might be a new combination treatment option. Moreover, GW inhibition of exosome production increased the concentration of doxorubicin in the tumor. Previous studies have shown that exosomes mediate drug resistance by reducing intracellular drug concentration in U937 cells, and it was found that GW4869 reduced drug export by inhibiting exosomes, thereby weakening cell resistance and enhancing drug efficacy [37]. This echoes our finding that GW4869 combined with doxorubicin can increase the intracellular drug concentration in tumor cells. We consider that the reason for this phenomenon may be that the inhibition of GW4869 on exosomes‐induced drug export and the reduction of angiogenesis in the tumor, and the normalization of blood vessels, thereby increasing drug entry and concentration.

Numerous studies have shown that tumor cell‐derived exosomes can promote tumor angiogenesis, growth, and metastasis [38, 39], with the underlying mechanism not fully understood. miRNA, lncRNA, circRNA, or some proteins in exosomes are the potential mediators [16, 40, 41] and blocking the corresponding molecules in exosomes can inhibit tumor angiogenesis and thus growth significantly [42, 43]. For example, Baoai Han et al. recently found that exosomes produced by high metastatic potential breast cancer cells are rich in EPHA2, which can promote angiogenesis and metastasis via AMPK signaling [42]. In a separate study, Chen demonstrated that exosomes from colorectal cancer (CRC) cells promoted vascular endothelial cell migration and tube formation. Suppressing circTUBGCP4 expression in CRC cell‐derived exosomes halted endothelial cell migration, tube formation, tip cell generation, and CRC metastasis [44]. Moreover, HeLa cell‐derived exosomes drive metastasis by inducing ER stress in endothelial cells, causing the breakdown of endothelial integrity through a mechanism independent of microRNAs [45]. We here identified that breast cancer cell‐derived exosomes could promote angiogenesis at least partially via promoting cell proliferation and systemically inhibiting exosome biogenesis emerges as a novel anti‐angiogenesis strategy.

Beyond being endocytosed by endothelial cells, tumor‐derived exosomes could also be delivered into the liver and spleen. The exosomes could also be functional in liver and spleen cells, and blocking tumor‐derived exosome biogenesis could also be beneficial for these tissues/organs. In addition, we believe that GW4869 would have side effects as normal cells also use exosomes to communicate. In this study, we did not see the obvious side effects mainly due to: (1) exosome biogenesis in normal cells is not totally inhibited; (2) The role of exosomes in normal cells could be complemented by other ways; (3) The toxicity examination methods are not sensitive enough; (4) The toxicity of GW4869 was covered by doxorubicin as suggested.

It is also important to note that GW4869 only inhibits ESCRT‐independent pathway, and exosomes biogenesis could be partially inhibited in our study. Development of strategies to block exosome biogenesis totally in tumor cells would pave the way for a better combination strategy. Mesenchymal stem cells (MSCs) not only have a strong ability of proliferation and differentiation, but also are easy to be cultured and manipulated, therefore, MSC‐derived exosomes have emerged as a promising avenue for pioneering cell‐free or targeted therapeutic strategies across a spectrum of ailments [46, 47]. Recent studies have also demonstrated that plant‐derived EVs (P‐EVs) can be engineered as drug‐loaded carriers to enhance the method and effectiveness of drug delivery. This is attributed to their biocompatibility, biodegradability, and abundant availability across various plant sources [48]. Loading GW4869 with the engineered exosomes or native exosomes having tumor targeting capacity would be a promising strategy.

### 4.1 Limitations of the Study

In this study, we have not identified the specific molecules in the exosomes that play the angiogenesis‐promoting role. Further studies are needed to identify the specific molecules and the underlying mechanism. In addition, no clinically relevant studies were included, which also need further investigation.

## Author Contributions

**Jindi He:** data curation (equal), methodology (equal), validation (equal), visualization (equal), writing – original draft (equal). **Fengyi He:** data curation (equal), methodology (equal), visualization (equal), writing – review and editing (equal). **Qinlian Yang:** data curation (equal), methodology (equal), validation (equal), visualization (equal). **Qiuyun Li:** conceptualization (equal), funding acquisition (equal), resources (equal), supervision (equal), writing – review and editing (equal).

## Disclosure

All animal experiments in this study were approved by the Institutional Animal Experiment Administration Committee of the Fourth Military Medical University.

## Ethics Statement

The authors have nothing to report.

## Consent

The authors have nothing to report.

## Conflicts of Interest

The authors declare no conflicts of interest.

## Supporting information


**Figure S1.** Distribution of exosomes in indicated organs in vivo.

**Figure S2.** Toxicity and side effects of Dox in combination with GW in mice.

## Data Availability

This research did not produce novel reagents. The data presented in this paper will be provided by the corresponding author upon request. Original code was not included in this paper. For any further details necessary to re‐examine the data presented in this study, interested parties can obtain them from the corresponding author upon making a reasonable request.
