# Supplementary figures and images for: Blockade of Exosome Release Sensitizes Breast Cancer to Doxorubicin via Inhibiting Angiogenesis
Source: Cancer Med. 2025 Apr 18;14(8):e70785. doi: 10.1002/cam4.70785 (PMC12007427; doi:10.1002/cam4.70785)

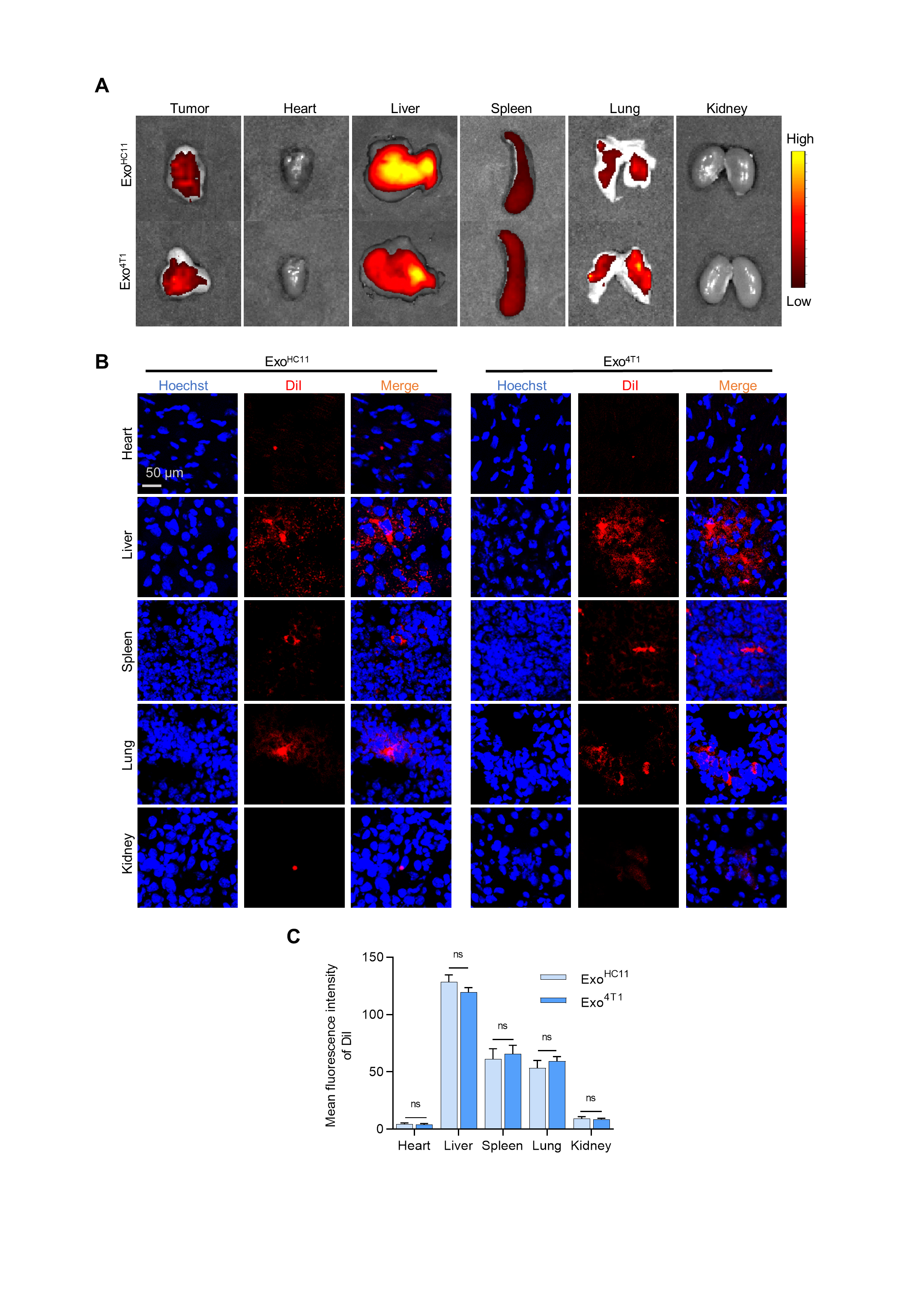

Supplement: Supplementary file 1 — Figure S1. Distribution of exosomes in indicated organs in vivo. [file CAM4-14-e70785-s002.tif]

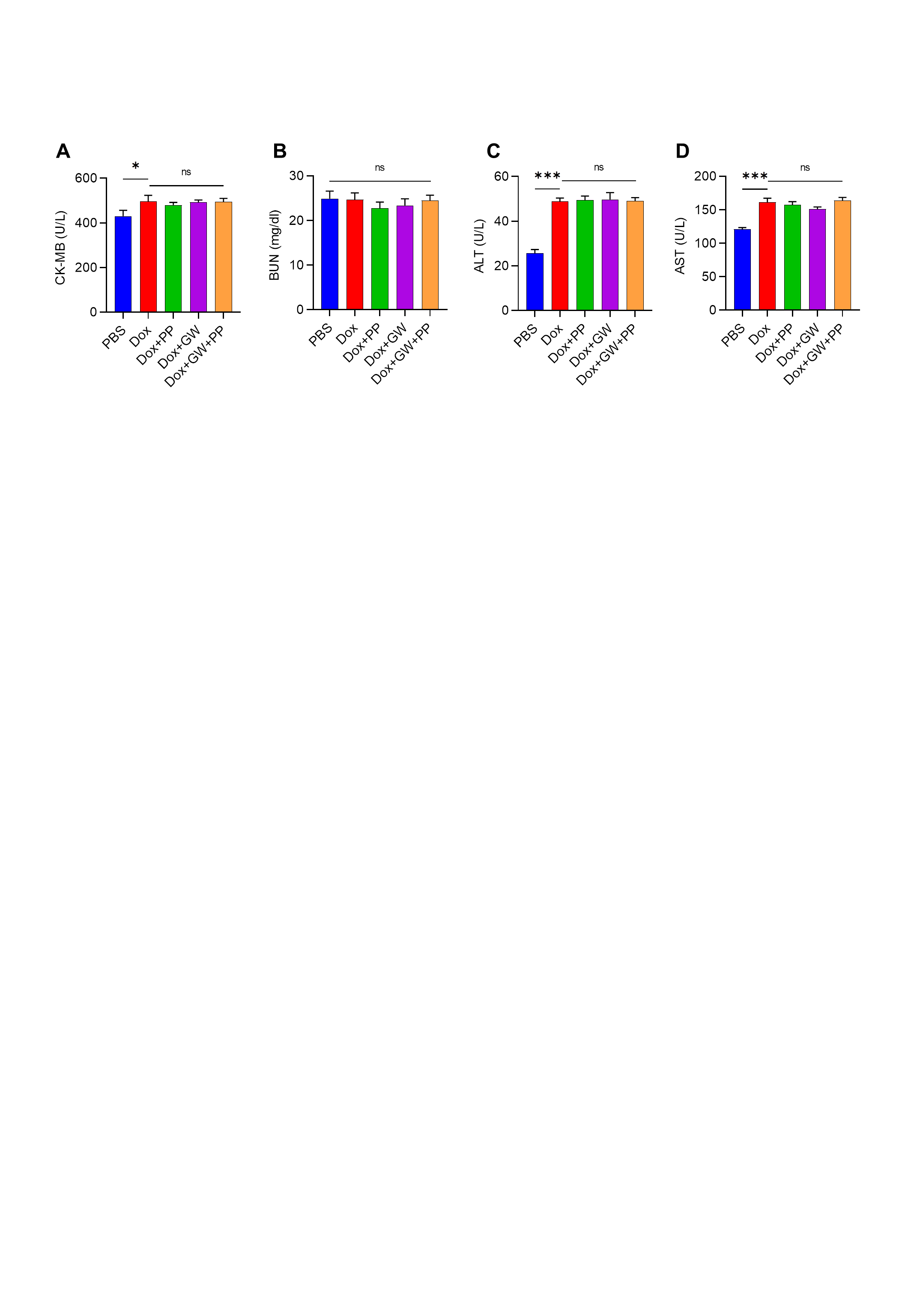

Supplement: Supplementary file 2 — Figure S2. Toxicity and side effects of Dox in combination with GW in mice. [file CAM4-14-e70785-s001.tif]
